# Xanthine Oxidase Activity in Type 2 Diabetes Mellitus Patients with and without Diabetic Peripheral Neuropathy

**DOI:** 10.1155/2016/4370490

**Published:** 2016-11-14

**Authors:** Dijana J. Miric, Bojana M. Kisic, Snezana Filipovic-Danic, Rade Grbic, Ilija Dragojevic, Marko B. Miric, Dragana Puhalo-Sladoje

**Affiliations:** ^1^Institute of Biochemistry, Medical Faculty, University of Pristina, Kosovska Mitrovica, Serbia; ^2^Clinics for Neurology and Psychiatry, Medical Faculty, University of Pristina, Kosovska Mitrovica, Serbia; ^3^Clinics for General and Orthopedics Surgery, Medical Faculty, University of Pristina, Kosovska Mitrovica, Serbia; ^4^Biochemical Laboratory, Medical Faculty, University of East Sarajevo, Foca, Bosnia And Herzegovina

## Abstract

This study investigated the relationship between serum xanthine oxidase (XOD) activity and the occurrence of diabetic peripheral neuropathy (DPN) in type 2 diabetes mellitus (T2DM) patients. Serum XOD activity, ischemia-modified albumin (IMA), uric acid (UA), albumin, glycated hemoglobin (HbA1c), advanced glycation end products (AGE), total free thiols, atherogenic index of plasma (AIP), and body mass index (BMI) were measured in 80 T2DM patients (29 with and 51 without DPN), and 30 nondiabetic control subjects. Duration of diabetes, hypertension, medication, and microalbuminuria was recorded. Serum XOD activities in controls, non-DPN, and DPN were 5.7 ± 2.4 U/L, 20.3 ± 8.6 U/L, and 27.5 ± 10.6 U/L (*p* < 0.01), respectively. XOD activity was directly correlated to IMA, UA, BMI, HbA1c, and AGE, while inversely correlated to serum total free thiols. A multivariable logistic regression model, which included duration of diabetes, hypertension, AIP, HbA1c, UA, and XOD activity, revealed HbA1c [OR = 1.03 (1.00–1.05); *p* = 0.034] and XOD activity [OR = 1.07 (1.00–1.14); *p* = 0.036] as independent predictors of DPN. Serum XOD activity was well correlated to several other risk factors. These results indicate the role of XOD in the development of DPN among T2DM patients.

## 1. Introduction

Diabetic peripheral neuropathy (DPN) is a late-stage microvascular complication that develops in nearly 50% patients during the course of type 2 diabetes mellitus (T2DM), affecting particularly low extremities. DPN is characterized by irreversible nerve structural and functional changes due to demyelination, axonal atrophy, and diminished regenerative potential, clinically presenting as a symmetric chronic pain, paraesthesia, and sensory loss. The development of DPN often worsens the quality of life [1], and increases the risk of cardiovascular morbidity, foot ulcerations, amputations, and overall mortality [2].

Although exact mechanisms are not fully understood, DPN is generally regarded as the consequence of chronic hyperglycemia-induced endothelial dysfunction, resulting in impaired endoneurial blood flow, ischemia, and nerve hypoxia [3]. Metabolic-vascular interactions in DPN are however highly complex and include diverse molecular pathways, like activation of protein kinase C and polyol metabolism, glycation and glycoxidation, low-grade inflammation, and excessive formation of reactive oxygen species (ROS), such as superoxide and hydrogen peroxide [4]. In vascular compartment these ROS can react with intrinsic vasodilator nitric oxide, and while the fall of nitric oxide levels would cause vasoconstriction, the resulting peroxynitrite could trigger the downstream events leading to nerve damage [5].

Because normal oxygen supply is fundamental for all tissues, impaired vasodilation is often associated with cellular energy crisis and increased breakdown of purine nucleotide to uric acid (UA) via xanthine oxidoreductase. Under physiological conditions this enzyme functions as dehydrogenase, but during hypoxia or after limited proteolysis it is converted to oxidase form (XOD). Unlike dehydrogenase, XOD more readily generates ROS able to impair vascular relaxation. In diabetic patients, hyperglycemia-induced endothelial dysfunction is chronic and not limited solely to the blood vessels irrigating nerve tissue but to variable extent occurs throughout the body. Moreover, XOD has been previously linked to oxidative damage in diabetes [6, 7] and diabetic cataract [8], as well as metabolic syndrome and its cardiovascular complications [9], and the role of XOD was also proposed in an experimental model of diabetic neuropathy [10].

As a molecular fingerprint of imminent hypoxia, serum UA was reported higher in T2DM than in nondiabetic subjects, especially in those with DPN [11]. During hypoxia, circulating serum albumin can undergo conformational changes at N-terminus aspartyl-alanyl-hystidyl-lysine sequence, which decrease its natural ability to bind cobalt and several other transition metal ions. The resulting ischemia-modified albumin (IMA) was originally associated with hypoxic conditions during myocardial ischemia [12] but was thereafter reported also in T2DM [13] and some diabetic complications [14]. However, there are missing data regarding serum XOD activity or IMA in DPN. Currently, there is no effective therapy to cure DPN, and even with good glycemic control the chance to develop DPN is relatively high among T2DM patients. Given that XOD could be a significant source of ROS in vascular compartment causing endothelial dysfunction, and a possible therapeutic target, this study was aimed at investigating the relationship between XOD and DPN in T2DM patients.

## 2. Materials and Methods

### 2.1. Study Participants

This study enrolled 80 patients previously diagnosed with T2DM, both sexes, who attended our local Clinical Hospital Center on regular basis for diabetes control. Not included were patients with significant motor deficits, recent cardiovascular or cerebrovascular events, overt renal or hepatic diseases, and retinopathy and those with foot ulcerations, amputations, and recent inflammatory disease or with known autoimmune or malignant disease. Excluded were also patients treated with alpha-lipoic acid or allopurinol. The control group was consisted of 30 age- and sex-matched subjects without DM, recruited from medical stuff and their relatives, who met the same exclusion criteria. This study was conducted in accordance with the Declaration of Helsinki, after informed consent from patients was provided. The institutional review board of the Medical Faculty Pristina (Kosovska Mitrovica) has approved this study.

DPN was defined as symmetrical sensorimotor polyneuropathy, diagnosed using a simplified scoring system for bedside examination, the Diabetic Neuropathy Symptom (DNS) score [15], followed by Michigan Neuropathy Screening Instrument (MNSI) scoring system. All subjects with DNS score > 1 were further assessed using the MNSI. The MNSI consists of two parts: the first part is a 15-item “yes or no” questionnaire about history of sensory and motor dysfunction (pain, temperature sensitivity, tingling, numbness, cramps, muscle weakness, feet ulcers or cracks, and amputation). The second part of MNSI encompasses examination of foot skin appearance, inspection for foot ulcers, examination of ankle reflexes, vibration perception testing using a 128-Hz tuning fork test at the great toe, and fine touch sensation testing using a 10 g Semmes-Weinstein monofilament applied on the plantar sites of each foot. DPN was defined as MNSI questionnaire ≥ 7 or MNSI examination score > 2 [16].

Hypertension was defined as having systolic blood pressure ≥ 140 mmHg or diastolic blood pressure ≥ 90 mmHg or being on antihypertensive medication. Microalbuminuria, as a marker of incipient kidney damage, was defined as urinary albumin-to-creatinine ratio of 30–300 mg/g [17]. Body mass index (BMI) was calculated as the ratio of body weight (kg) and square of body height (m^2^).

### 2.2. Biochemical Methods

Venous blood was taken after an overnight fasting into vacutainer tubes without or with anticoagulant (EDTA) to obtain serum, plasma, or whole blood samples. The first morning urine sample taken into sterile urine containers during two months in three nonconsecutive days was provided for determination of urinary albumin and creatinine.

Serum XOD activity was measured according to the method of Roussos [18], as described earlier [8]. XOD activity was calculated after correction for preexisting uric acid, using molar absorbance of uric acid at *λ* = 293 nm, of *ε* = 1.26 × 10^4^ L × M^−1^ × cm^−1^. One unit of XOD activity was defined as 1 *μ*mol/min uric acid formed at 37°C. Serum IMA was measured using a colorimetric method described by Bar-Or et al. [12]. Results were expressed as units per milliliter serum (IU/mL). Concentration of total serum thiols, as an indicator of protein oxidative damage, was measured using Ellman's reagent [19]. Concentration of serum AGE was determined spectrofluorometrically by the method of Kalousová et al. [20] and expressed as relative fluorescence units (RFU). Concentration of advanced oxidation protein products (AOPP), as marker of chronic oxidative albumin damage, was determined from lipid-depleted plasma samples to avoid interference with lipid status, by the method of Anderstam et al. [21]. The results were expressed as *μ*mol/L Chloramine-T equivalents. Serum XOD, AGE, and AOPP were assessed from aliquoted samples (0.5 mL) kept at −80°C.

Concentrations of serum and urinary creatinine, serum total proteins, albumin, triglycerides, total cholesterol, HDL-cholesterol, UA, and blood glycated hemoglobin (HbA1c) were measured on Cobas Integra 400 biochemical analyzer, using standard protocols. Urinary albumin was determined using Tinaquant Albumin Gen.2 assay kit (Roche Diagnostics GmbH, Mannheim, Germany). The average of urinary albumin excretion was calculated for each patient from results of three nonconsecutive urine samples. Concentration of LDL-cholesterol was calculated using Friedewald's formula. Atherogenic index of plasma (AIP), as a surrogate marker of atherogenic dyslipidemia, was calculated according to Dobiasova, as the logarithm of triglycerides to HDL-cholesterol concentration ratio [22].

### 2.3. Statistical Methods

Data analyses were performed using MedCalc Software package (Mariakerke, Belgium). Frequency distribution and homogeneity of variance were tested by Kolmogorov-Smirnov test. Data were presented as either arithmetic mean ± SD, frequencies (*f*), or median and 95% confidence interval of the median. Differences between groups were tested by ANOVA and Student's independent samples *t*-test, or chi-square test, where appropriate. Correlation analysis was accomplished by calculation of Spearman's coefficient. The relationship between DPN, XOD, and other risk factors was investigated by multivariable logistic regression analysis. Statistically significant finding was considered if *p* < 0.05.

## 3. Results

Basic demographical, clinical, and biochemical findings in controls and T2DM patients are presented in Table 1. Of 80 T2DM patients enrolled in the study, 29 were with DPN; the remaining (*n* = 51) comprised the non-DPN group. There were no significant differences between control and groups with DM, in terms of age and sex distribution, smoking status, presence of hypertension, serum creatinine, and total protein levels (Table 1). Twenty-five T2DM patients were with microalbuminuria, having urinary albumin to creatinine ratio within the range of 30–300 mg/g. Patients with T2DM had significantly higher BMI than control subjects and were rather overweight (*n* = 31) or obese (*n* = 26).

Concentrations of fasting blood glucose, total cholesterol, HDL-cholesterol, LDL-cholesterol, triglycerides, and AIP significantly differed from controls in both groups with T2DM (Table 1). In comparison to non-DPN group, concentration of HDL-cholesterol was significantly lower in DPN group.

In the overall sample the median XOD activity was 16.4 U/L (95% CI 13.8–20.4). Serum XOD activity and IMA concentration were higher in T2DM patients than in control subjects and significantly differed between DPN and non-DPN groups (Table 2). Serum albumin and total thiols were lower in DM patients than in controls and differed between DPN and non-DPN groups. Compared to controls, HbA1c and serum AGE were increased in DM patients, being higher in DPN than in non-DPN group; the occurrence of DPN also affected serum UA concentrations. Serum AOPP was higher in T2DM patients than in controls, but the difference between diabetic groups was insignificant (Table 2).

In patients with T2DM, serum XOD activity was directly correlated to BMI, the presence of hypertension, and levels of HbA1c, AGE, IMA, and UA and inversely correlated to serum albumin and total thiol groups concentrations (Table 3). Correlations between XOD activity and age, duration of diabetes, AIP, AOPP, and microalbuminuria were not significant.

We further analyzed the association between several clinical and biochemical variables and the occurrence of DPN. Included were duration of diabetes, HbA1c, AIP, hypertension, serum XOD, and UA levels. The multivariable logistic regression analysis (*B* = −5.014; chi-square = 20.023; *p* = 0.0027) clearly revealed HbA1c and serum XOD activity as independent predictors of DPN, whereas the influence of diabetes duration was of the borderline significance (Table 4).

## 4. Discussion

The major finding of the present study was significant elevation of serum XOD activity in patients with T2DM and an independent association between XOD activity and the occurrence of DPN. Serum XOD activity was well correlated to the levels of IMA and some other biomarkers of increased ROS formation. Furthermore, XOD activity was directly correlated to several risk factors relevant for the development of DPN, including hypertension, higher BMI, and concentrations of HbA1c and UA, indicating the role of XOD in the development of DPN.

XOD is a rate-limiting enzyme of purine catabolism to UA, during which high quantities of ROS are produced. We observed that the formation of ROS was specifically increased in DPN group, documented as decreased serum total free thiols and increased levels of oxidatively damaged molecules, like IMA and AGE, accompanied by higher concentration of serum UA (Table 2). These findings are consistent with the concept that increased ROS formation can contribute to the development of DPN [4, 23].

Serum total free thiol groups essentially originate from a single 34 cystein residue of albumin, which is abundantly present blood protein. Acting as nonenzymatic antioxidant and reducing agent these free thiols are rapidly oxidized after exposure to ROS, forming mixed disulfides and related sulfur-containing acids. In one previous study the loss of total serum thiols was found to correspond to the severity of diabetic microvascular complications and the development of DPN [24], and our current results support that finding.

There was also a significant increase in serum IMA concentrations in T2DM, especially in DPN group, in our study. Several other studies reported elevated serum IMA levels in diabetes, correspondingly to the presence or severity of complications, including peripheral arterial disease [14]. We observed that IMA concentrations were positively correlated to XOD activity, indicating that oxidative damage of circulating biomolecules in DPN can be, at least partly, inflicted by XOD-derived ROS. Although exact chemical mechanisms of IMA formation are still a matter of debate, it is generally accepted that IMA is formed due to oxidative damage of serum albumin N-terminus [14], specifically in hypoxic conditions, such as those created by dysfunctional blood vessels. In accordance to that, IMA is now regarded as a biomarker of widespread endothelial damage in a variety of pathological states, including T2DM [13]. However, in the absence of other biomarkers of endothelial damage these results should be cautiously interpreted. Besides IMA, the occurrence of DPN was associated with higher serum UA and lower albumin levels, which are known risk factors of DPN. Also, 31% of patients included in the study were microalbuminuric; thus the coexistence of decreased UA elimination rate and increased urinary albumin excretion rate as confounding factors cannot be excluded.

Still, increased serum levels of IMA and UA have been regarded as biomarkers widespread endothelial dysfunction in a variety of pathological states, including T2DM [13, 25]. Tissues irrigated by dysfunctional vessels inevitably suffer hypoxic and even anoxic conditions, which promote the transition of xanthine dehydrogenase to XOD form. Within vascular compartment the enzyme is found mostly at XOD form, attached to endothelial cell surface via sulfated glycosaminoglycan-rich receptors, and as a free enzyme in the blood. Both bound and free XOD can generate ROS able to modify circulating blood and vascular wall constituents, such as albumin and endothelium-derived nitric oxide. The reaction of nitric oxide and superoxide anion radical diminishes nitric oxide levels and yields peroxynitrite, which can act as toxic mediator inducing nerve injury [5]. At the same time, diminished availability of nitric oxide would exacerbate endothelial dysfunction, since it is implicated in regulation of vascular tone, as well as in prevention of platelet adhesion and aggregation and leukocytes adhesion to the vascular wall. Moreover, augmented serum XOD activity has been already linked to increased ROS formation in metabolic syndrome and its cardiovascular complications, clinical and experimental diabetes, and diabetic ocular complications [6–9]. To the best of our knowledge this is the first report of XOD in T2DM with DPN.

The majority of vascular XOD is believed to be of hepatic origin, nonspecifically released into the blood, particularly under hyperglycemic conditions [6]. It was therefore not surprising that HbA1c levels were directly correlated to XOD activity in our and some previous studies [6, 7], thereby confirming that glycemic control plays critical role in modulating XOD presence/activity within vascular compartment. Besides HbA1c, serum XOD activity was also correlated to AGEs, which are chemically heterogeneous products of protein nonenzymatic glycation and subsequent oxidation reactions. AGEs not only are biomarkers of glycoxidative protein damage but also act as toxic mediators after binding to specific receptors, like multiligand RAGE or macrophage scavenger receptors. In peripheral nerves RAGE are primarily expressed in endothelial and Schwann cells. It is of note that AGE-RAGE interaction facilitated endoneurial vascular dysfunction in peripheral nerves, leading to microangiopathy [26].

Etiology of DPN is however highly complex, since diverse though interrelated metabolic lifestyles and genetic factors can be involved [6]. For example, a prolonged ischemia and obesity can be both present in patients with T2DM and associated with higher levels of serum UA [27]. In fact, one recent study demonstrated that, beyond purine metabolism, XOD also plays a role in differentiation of adipocytes acting as a regulator of the nuclear receptor peroxisome proliferator-activated receptor *γ* activity, which is the key factor controlling the induction and maintenance of adipogenesis [28]. In the present study, serum XOD activity was correlated to BMI as well as to UA levels (Table 3). One probable explanation could be a high prevalence of overweight (38.7%) and obese (23.5%) diabetics, and since XOD is abundantly expressed in adipocytes it might cause increased secretion of UA from adipose tissue, especially during ischemia [29]. These results are consistent with those of Feoli et al., who reported a similar relationship in metabolic syndrome [9].

It has been previously shown that ROS formed in chronic hyperglycemia may induce unmyelinated and myelinated nerve fibers loss and impairment of endoneurial blood flow, resulting in nerve conduction velocity abnormalities [23]. Histologically, DPN is characterized by accumulation of macrophages in perivascular lesions [30]. Upon activation, macrophages can produce high quantities of ROS via XOD, thereby causing local endothelial dysfunction, further impairment of endoneurial blood flow, and myelin degeneration. Overexpression of XOD is actually necessary step for macrophages activation and regulation of proinflammatory mediators and chemokines secretion [31, 32]. Furthermore, ROS generated by local macrophages via XOD may induce nerve axonal loss and demyelination, as demonstrated in a murine model of neuroinflammation [33].

Because of high content of polyunsaturated fatty acids nervous tissue is exceptionally susceptible to ROS; thus tight control of prooxidant enzyme activity is of great importance. Previously, Inkster et al. reported that inhibition of XOD with allopurinol, a structural analogue of hypoxanthine with both hypouricemic and anti-inflammatory effects, was able to prevent the loss of motor and sensory conduction velocity in an animal model of diabetes [10]. Their study demonstrated that inhibition of XOD improved blood flow in sciatic and cervical nerve ganglion, thereby suggesting that upregulated XOD present in nerve microvasculature, including endothelium and perivascular space, can cause neurovascular dysfunction leading to diabetic neuropathy. Allopurinol also prevented cardiac ischemia and impaired relaxation in an experimental model of insulin resistance [34], normalized endothelial function in T2DM patients with mild hypertension [35], alleviated oxidative injury and improved cardiovascular functions in diabetics in several intervention studies [36], and acted antinociceptive against various noxious stimuli in mice [37]. On the other side, peripheral neuropathy can be a side effect of allopurinol treatment, and this drug failed to prevent the progression of DPN in type 1 diabetes mellitus patients with mild-to-moderate cardiovascular autonomic neuropathy [38].

Nonetheless, the current study has demonstrated that augmented XOD activity was present within vascular compartment in T2DM patients diagnosed with DPN. Moreover, serum XOD activity was closely correlated to risk factors relevant for the development of DPN, including poor glycemic control, obesity, and serum IMA and UA levels, which are considered as markers of generalized endothelial dysfunction in diabetics. Our results therefore indicate that upregulated XOD can be an additional source of ROS in vascular compartment, implicated in chronic oxidative injury and endothelial dysfunction in T2DM patients with poor glycemic control, contributing overtime to the development of DPN.

## Figures and Tables

**Table 1:** Basic demographical and clinical parameters in control subjects and type 2 diabetes mellitus patients with and without diabetic peripheral neuropathy.

	Control subjects (*n* = 30)	Type 2 DM patients	
		Non-DPN (*n* = 51)	DPN (*n* = 29)
Age (years)	60.3 ± 8.7	61.5 ± 9.3	62.9 ± 7.5
Gender (male/female; *n*)	13/17	20/31	13/16
BMI (kg/m^2^)	25.3 ± 4.1	29.3 ± 4.1^*∗*^	28.4 ± 4.5^*∗*^
Diabetes duration (years)	NA	5.83 ± 2.56	6.39 ± 2.98
Microalbuminuria (yes/no; *n*)	NA	15/36	10/19
uACR (mg/g)	NA	15.6 (11.8–20.7)	23.4 (15.6–35.2)
Hypertension (yes/no; *n*)	13/17	32/19	18/11
Current smokers (yes/no; *n*)	11/19	24/27	9/20
Antidiabetic medication			
(i) Oral antidiabetic drugs (*n*)		37	18
(ii) Oral antidiabetic drugs + insulin (*n*)	NA	10	8
(iii) Insulin (*n*)		4	3
Fasting blood glucose (mmol/L)	5.07 ± 1.19	7.34 ± 2.51^*∗*^	8.07 ± 3.04^*∗*^
Total cholesterol (mmol/L)	5.86 ± 1.05	6.81 ± 1.53^*∗*^	6.64 ± 1.76^*∗*^
HDL-cholesterol (mmol/L)	1.74 ± 0.56	1.47 ± 0.41^*∗*^	1.23 ± 0.41^*∗*‡^
LDL-cholesterol (mmol/L)	2.60 ± 0.87	3.88 ± 1.32^*∗*^	4.01 ± 1.45^*∗*^
Triglycerides (mmol/L)	2.16 ± 0.74	2.85 ± 1.04^*∗*^	2.77 ± 0.95^*∗*^
AIP [log (triglycerides/HDL)]	0.114 ± 0.229	0.270 ± 0.229^*∗*^	0.366 ± 0.238^*∗*^
Serum creatinine (*μ*mol/L)	76.3 ± 10.4	77.5 ± 10.7	77.0 ± 11.9
Total proteins (g/L)	76.9 ± 3.6	75.3 ± 3.1	75.9 ± 3.7

Data are presented as mean values ± standard deviation, frequencies (*n*), or geometric mean and 95% confidence interval of the mean (in parenthesis). Differences between groups were tested by one-way ANOVA, Student's independent samples *t*-test, or chi-square test. DPN: diabetic peripheral neuropathy; BMI: body mass index; NA: not applicable; uACR: urinary albumin to urinary creatinine ratio; AIP: atherogenic index of plasma.

^*∗*^ *p* < 0.05 versus control group; ^‡^ *p* < 0.05 versus non-DPN group.

**Table 2:** Serum XOD activity, uric acid, IMA, and other biomarkers of protein modifications in control subjects and type 2 diabetes mellitus patients with and without diabetic peripheral neuropathy.

	Control subjects (*n* = 30)	Type 2 DM patients	
		Non-DPN (*n* = 51)	DPN (*n* = 29)
XOD (U/L)	5.7 ± 2.4	20.3 ± 8.6^*∗*^	27.5 ± 10.6^*∗*‡^
IMA (IU/mL)	28.5 ± 8.7	34.7 ± 12.4^*∗*^	40.3 ± 11.8^*∗*‡^
Albumin (g/L)	44.1 ± 2.4	43.4 ± 2.2	42.1 ± 2.2^*∗*‡^
HbA1c (mmol/mol)	35.2 ± 11.8	59.2 ± 22.7^*∗*^	72.7 ± 22.8^*∗*‡^
Uric acid (*μ*mol/L)	246 ± 70	268 ± 72	298 ± 68^*∗*^
Total thiols (*μ*mol/L)	541 ± 94	407 ± 118^*∗*^	294 ± 86^*∗*‡^
AGE (RFU)	6.41 ± 1.19	7.26 ± 1.44^*∗*^	8.45 ± 2.17^*∗*‡^
AOPP (*μ*mol/L Chloramine-T)	38.7 ± 9.4	63.7 ± 23.7^*∗*^	65.3 ± 26.0^*∗*^

Data are presented as mean values ± standard deviation. Differences between groups were tested by one-way ANOVA and post hoc Student's *t*-test. XOD: xanthine oxidase; IMA: ischemia-modified albumin; AGE: advanced glycation end products; AOPP: advanced protein oxidation products; RFU: relative fluorescence units.

^*∗*^ *p* < 0.05 versus control group; ^‡^ *p* < 0.05 versus non-DPN group.

**Table 3:** Relationships between serum XOD activity and various clinical and biochemical variables in type 2 diabetes mellitus.

	XOD (U/L)	
	Spearman's rho	*p* value
Age (years)	0.192	0.089
Diabetes duration (years)	0.120	0.287
HbA1c (mmol/mol)	0.244	0.030
BMI (kg/m^2^)	0.275	0.015
AIP [log (triglycerides/HDL)]	0.165	0.144
Uric acid (*μ*mol/L)	0.250	0.026
Albumin (g/L)	−0.299	0.002
Total thiol groups (*μ*mol/L)	−0.267	0.018
AGE (RFU)	0.260	0.021
AOPP (*μ*mol/L Chloramine-T)	0.020	0.857
IMA (IU/mL)	0.227	0.044
Hypertension (yes versus no)	0.229	0.042
Microalbuminuria (yes versus no)	0.188	0.095

Correlation analysis was accomplished by calculation of Spearman's nonparametric coefficient (rho) in 80 type 2 DM patients.

**Table 4:** Multivariable logistic regression analysis for association with diabetic peripheral neuropathy.

Independent predictors	*β*	Std. error	*p* value	OR (95% CI)
Diabetes duration (years)	0.182	0.096	0.058	1.20 (0.99–1.45)
AIP [log (triglycerides/HDL)]	2.293	1.318	0.082	9.91 (0.75–131.17)
HbA1c (mmol/mol)	0.026	0.012	0.034	1.03 (1.00–1.05)
Serum uric acid (*μ*mol/L)	0.006	0.004	0.165	0.99 (0.98–1.00)
Hypertension (yes versus no)	0.687	0.564	0.223	1.99 (0.66–6.00)
XOD (U/L)	0.067	0.032	0.036	1.07 (1.00–1.14)
